# Augmented pain inhibition and higher integration of pain modulatory brain networks in women with self-injury behavior

**DOI:** 10.1038/s41380-022-01639-y

**Published:** 2022-06-13

**Authors:** Maria Lalouni, Jens Fust, Johan Bjureberg, Gránit Kastrati, Robin Fondberg, Peter Fransson, Nitya Jayaram-Lindström, Eva Kosek, Clara Hellner, Karin B. Jensen

**Affiliations:** 1 Department of Clinical Neuroscience, Karolinska Institutet, Stockholm, Sweden; 2 Centre for Psychiatry Research, Department of Clinical Neuroscience, Karolinska Institutet & Stockholm Health Care Services, Region Stockholm, Stockholm, Sweden; 3 Department of Psychology, Stanford University, Stanford, CA USA; 4 Department of Surgical Sciences, Uppsala University, Uppsala, Sweden

**Keywords:** Psychiatric disorders, Neuroscience

## Abstract

Individuals who engage in nonsuicidal self-injury (NSSI) have demonstrated insensitivity to pain compared with individuals without NSSI. Yet, the neural mechanisms behind this difference are unknown. The objective of the present study was to determine which aspects of the pain regulatory system that account for this decreased sensitivity to pain. In a case–control design, 81 women, aged 18–35 (mean [SD] age, 23.4 [3.9]), were included (41 with NSSI and 40 healthy controls). A quantitative sensory testing protocol, including heat pain thresholds, heat pain tolerance, pressure pain thresholds, conditioned pain modulation (assessing central down-regulation of pain), and temporal summation (assessing facilitation of pain signals) was used. Pain-evoked brain responses were assessed by means of fMRI scanning during thermal pain. NSSI participants showed a more effective central down-regulation of pain, compared to controls, assessed with conditioned pain modulation. The neural responses to painful stimulation revealed a stronger relation between nociceptive and pain modulatory brain regions in NSSI compared to controls. In line with previous studies, pressure and heat pain thresholds were higher in participants with NSSI, however, there were no correlations between pain outcomes and NSSI clinical characteristics. The augmented pain inhibition and higher involvement of pain modulatory brain networks in NSSI may represent a pain insensitive endophenotype associated with a greater risk for developing self-injurious behavior.

## Introduction

The sensation of pain is inherently aversive and shapes us to avoid situations that cause harm to our bodies. Yet, there are individuals who deliberately induce pain by self-injury. Nonsuicidal self-injury (NSSI) is defined as intentional self-inflicted tissue damage, performed without suicidal intent, and for purposes that are not socially sanctioned [1]. NSSI is highly prevalent in adolescents and young adults [2] and is associated with high psychiatric comorbidity [3, 4] and an increased risk for suicide [5, 6].

Even though NSSI results in tissue damage, many individuals who self-injure report feeling little or no pain while they hurt themselves [7–9]. One study revealed that individuals who did not experience pain during NSSI reported twice as many suicide attempts, compared with individuals who experienced pain during NSSI [10]. Sensitivity to physical pain has thus been suggested [11] as one of the barriers that may hinder the development of NSSI in most humans, and a better understanding of aberrant pain perception may help determine who is at risk of developing NSSI. Previous data suggest that pain insensitivity in individuals with NSSI is not limited to self-injury per se, as laboratory studies have displayed heightened pain thresholds and pain tolerance [12, 13] Borderline symptomatology [12, 14], self-critical cognitive style [15, 16], and dysregulation of the opioid system [17, 18] have been proposed as underlying causes to the pain insensitivity, but results are inconclusive.

Laboratory pain testing, or Quantitative Sensory Testing, can be used to assess different aspects of pain processing. For example, a pronociceptive pain profile (low inhibition/high facilitation of pain) is believed to be a risk factor for developing chronic pain [19]. Conversely, individuals with NSSI may represent an antinociceptive pain profile (high inhibition/low facilitation of pain), resulting in lower sensitivity to pain. To date, there have been few attempts to quantify pain modulation in individuals with NSSI [20] and the results are inconclusive.

The aim of the present study was to determine the mechanisms of pain regulation in a well-characterized sample of women with NSSI compared to matched controls. We hypothesized that NSSI participants would display enhanced conditioned pain modulation (pain inhibition) and decreased temporal summation (pain facilitation). A further aim of the study was to assess the relationship between pain regulation and clinical characteristics, as well as differences in brain responses to painful stimuli.

## Methods

This case–control study was approved by the Regional Ethical Review Board in Stockholm (2018/1367-31/1) and pre-registered on Open Science Framework May 27^th^ 2019 (https://osf.io/gujwt/). Deviations from pre-registration are reported at https://osf.io/ws63d/. The fMRI analysis was explorative and not pre-registered.

### Setting

All study visits took place at the MR Center, Karolinska University Hospital in Stockholm, Sweden, between May 2019 and August 2020, with a brief disruption during the first wave of the Covid-19 outbreak from mid-March to the end of May 2020. This study is part of larger research project, investigating women with NSSI.

### Participants

Based on a recent meta-analysis by Koenig et al. [12], we estimated that we needed 29 participants in each group to achieve 80% power to detect a difference in pain threshold between NSSI participants and controls. We decided to recruit 40 participants to be able to detect the potentially smaller differences between groups in the other pain tests. Participants with NSSI (*n* = 41) and controls (*n* = 40) were recruited between April 2019 and June 2020, through flyers in waiting rooms at outpatient psychiatric clinics (NSSI) and advertisements in social media (NSSI and controls). The advertisement for controls was adjusted during the inclusion procedure so that age and educational level would match those of the NSSI participants. *General inclusion criteria were*: a) woman, b) 18–35 years, and c) right-handed. General exclusion criteria were: d) chronic inflammatory, autoimmune, or other somatic disorder requiring treatment, e) pain condition, f) contraindication for fMRI (e.g., metal implant, pregnancy, claustrophobia), g) suicide attempts during the last year, h) suicidal plans or acute risk for suicide. Specific inclusion criteria for participants with NSSI: i) self-injury ≥5 days during the last year. Specific exclusion criteria for controls: j) treatment for depression or anxiety. Participants with NSSI received a remuneration of $171 (1500 SEK) and controls received $137 (1200 SEK). Participants with NSSI received the higher amount because their participation included a visit to a psychologist (described under Study Procedure).

### Pain measures and equipment

The pain testing included:

1. *Heat pain thresholds and tolerance* were assessed with a thermal stimulator (MSA Thermal Stimulator Somedic, Hörby, Sweden) using a (30 × 30 mm) thermode.
2. *Pressure pain thresholds* were assessed with a handheld algometer (Somedic Algometer version II, Hörby, Sweden) with a 1 cm^2^ round rubber tip. The algometer records pressure in kilopascals (kPa) and was set to indicate when a speed of 50 kPa/s was used. An automatic pressure apparatus (APA Somedic, Hörby, Sweden), with identical characteristics as the algometer, was used for 5 persons in the NSSI group. This apparatus was later replaced with the algometer, due to technical difficulties.
3. *Conditioned pain modulation*. Pressure pain thresholds (kPa) applied with an algometer on the left calf was used as test stimulus and ischemic pain to the lower right arm was used as conditioning stimulus. The ischemic pain was induced with a blood pressure cuff connected to a cuff inflation system (Hokanson Inc, Bellevue, WA, USA) in combination with a handheld dumbbell (1 kg) that was flexed up and down (1 movement per second).
4. *Temporal summation* was assessed with PinPrick Stimulators (MRC Systems, Heidelberg, Germany). The PinPrick kit consists of seven stimulators, each with a flat contact area of 0.25 mm in diameter and weights between 8 mN and 512 mN. A stimulator that induced low pain, between 1/10 and 3/10 on a numerical rating scale (NRS), was individually tried out and subsequently used.

### fMRI

Magnetic resonance images were acquired with a 3T General Electric 750 MR scanner. Functional scans using T2*-weighted single-shot gradient echo planar imaging were collected, using the following parameters: repetition time/ echo time = 2000/30 ms, flip angle = 70°, field of view = 220 × 220 mm, matrix size = 72 × 72, 42 slices, slice thickness = 3 mm with a 0.5-mm gap, interleaved slice acquisition. Anatomical images were acquired with a high-resolution BRAVO 3D T1-weighted image sequence (1 × 1 × 1-mm voxel size, 176 slices).

### Questionnaires

Frequency and function of NSSI were assessed with the Functional Assessment of Self-Mutilation (FASM) [8, 9]. Borderline symptoms were assessed with the short version of the Borderline Symptom List (BSL-23) [21]. The Self-Rating Scale (SRS) was used to assess self-criticism [22] Emotion regulation was assessed with the brief 16 item version of the Difficulties with Emotion Regulation Scale (DERS-16) [23]. Suicidal behaviors were self-assessed online with the Self-Injurious Thoughts and Behaviors Interview-Short Form-Self Report (SITBI-SF-SR) [9, 24] NSSI frequency was measured by the question in FASM: “How many days have you engaged in the type of self-harm mentioned in the previous questions?”. Participants were asked to check one of four boxes: 1–4, 5–15, 15–50, 50-. Severe NSSI methods score was calculated by summing up the prevalence of self-reported use of a subset of NSSI methods in FASM (cutting/carving, burning, scraping, and erasing the skin), as defined by Lloyd-Richardson et al. [8]. NSSI recency was measured on visit 1 by the question: “How many days have passed since you last engaged in self-harm behavior?”. NSSI duration was measured by a question from FASM: “How old were you when you first harmed yourself in this way [described in the previous questions]?”

### Study procedure

Written informed consent was obtained from all subjects. The self-report questionnaires were assessed through a secure online data collection platform (BASS, eHealth Core Facility, Karolinska Institutet, Sweden). NSSI participants met a licensed psychologist once who assessed their potential risk of participating in the study and asked about their current psychiatric comorbidity. Pain tests were then assessed at two consecutive visits for both NSSI participants and controls. Participants were asked to withhold from as needed medication or drugs that could affect their pain regulation (eg, tranquilizers, NSAIDs, paracetamol) 24 h before the pain tests. However, if they urgently needed to take such medication, they could do so.

#### Visit 1 - Behavioral pain tests

**Fig. 1:**
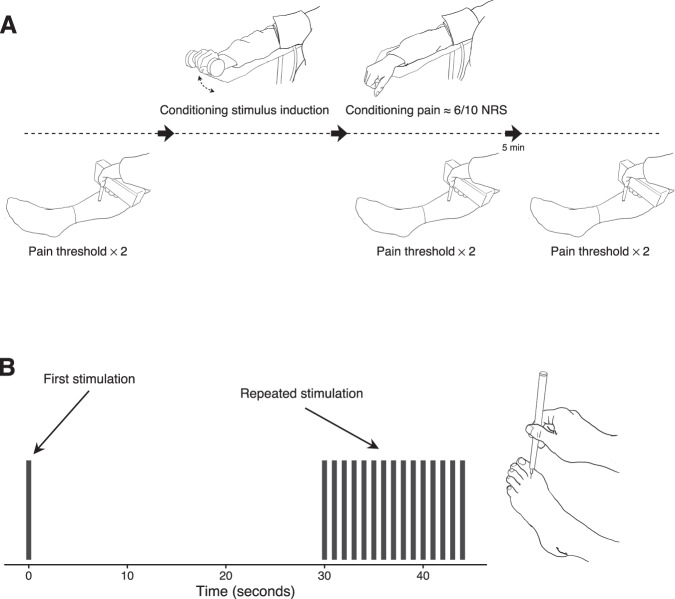
Methods. **A** Testing procedure for conditioned pain modulation. **B** Temporal summation. NRS numerical rating scale.

Heat pain threshold and tolerance were assessed with a thermode on the participants left calf using repeated heat stimuli (5 s long with an interstimulus interval of 30 s). Pain was rated on a continuous 0–10 scale (NRS). The participants were exposed to between 13–24 heat stimulations (depending on the pain sensitivity of the participant). The temperature never exceeded 50 °C, to avoid the risk of tissue damage (for a detailed description of the heat pain calibration procedure, see Supplementary A). Pressure pain thresholds were assessed with an algometer on the participants’ anterior, distal part of the left thigh (quadriceps femoris). Participants indicated when the pain threshold was reached by pressing a button. A total of 3 pressure stimulations were administered. During conditioned pain modulation pressure pain thresholds were assessed on the participants’ left calf twice before the conditioning stimulus was induced, twice during the conditioning stimulus, and twice after the conditioning stimulus was removed (Fig. 1A). The last two pressure pain thresholds were assessed to ensure that the participants’ pain sensitivity returned to baseline.

#### Visit 2 - Pain tests in the MR-scanner

During temporal summation, pain was induced with a PinPrick stimulator on the participants’ left foot, between the big toe and second toe (Fig. 1B). Pain was induced a) once, and b) 15 times (once per second), four times each with a break of 30 s between the four trials. Imaging data for temporal summation will be presented elsewhere. Individually calibrated heat pain (approximately NRS 5/10) was used to provide 30 s blocks of “pain on” (three blocks) and “pain off” (three blocks). A thorough heat calibration procedure had been employed to ensure that all participants were perceiving the same 5/10 NRS intensity pain during MRI scans and that the “pain off” temperature was perceived as non-painful. The heat pain was induced on the participants’ left calf with a thermode. Each block was preceded by a visual cue on a screen inside the MR-scanner (10 s duration) indicating if the block would include pain or not. After each stimulation the pain was rated on a 0–10 NRS.

### Statistical analysis

#### Behavioral data

Baseline variables for NSSI participants and controls were compared using χ^2^ for categorical variables and 2-tailed *t* tests for continuous variables. Pressure pain thresholds were defined as the mean kPa of three trials. Heat pain threshold and heat pain tolerance were calculated by fitting a linear regression to each participant’s pain ratings, with stimulus temperature as a predictor. Heat pain threshold was defined as the participant’s predicted NRS 1/10 and heat pain tolerance was defined as the participants predicted NRS 6/10. Comparisons between the two groups’ pain threshold and tolerance were made using Wilcoxon rank sum test. The conditioned pain modulation effect was defined as the difference between pressure pain thresholds, before and during the conditioning stimulus. A mixed effect model was fitted with conditioning stimulus (on/off), group (NSSI/controls) and the interaction between conditioning stimulus and group specified as fixed effects. As random effects, we used by-subject intercepts and by-subject slopes for conditioned stimulus. Temporal summation was assessed by the facilitation of pain, defined as the difference between the first pain rating and the maximum pain rating during the repeated stimulation. A mixed effects model, in which stimulus (first/max), group (NSSI/controls), and the interaction between stimulus and group was specified as fixed effects, and by-subject intercepts and by-subject slopes for stimulus was specified as random effects. Lastly, pain outcomes were correlated with self-report data of NSSI behavior and clinical characteristics using Kendall rank correlation coefficient. Because of the exploratory nature of these correlations, we did not correct for multiple comparisons. The statistical analyses were conducted in R [25] and Stata [26]. Mixed effect model was fitted R packages lme4 [27] and lmerTest [28].

#### Neuroimaging data

Data analyses were performed using Statistical Parametric Mapping 12 (SPM12) [29], and Matlab2014 (The MathWorks, Inc, Natick, MA). For preprocessing parameters, see Supplementary A. A first-level general linear model (GLM) was built for each individual and included the following regressors of interest: pain cue, pain-on, pain rating, no-pain cue, no-pain. Regressors of interest were convolved with the canonical hemodynamic response function. Six motion parameters were added as regressors of no interest. On the group level, one-sample *t*-tests were performed to determine brain activation patterns across all participants. Two-sample *t*-tests were used to determine differences in brain activation between groups. Statistical significance was considered at an initial statistical threshold of *P* < 0.05 FWE corrected over the entire brain, and then cluster-level *P* < 0.05 FWE corrected.

Post-hoc analyses were performed by applying two pain signatures aimed to characterize neural pain processing without the burden of multiple comparisons. The Neurologic Pain Signature (NPS) is derived from a machine-learning protocol and restricts the search volume to pain-specific brain regions [30]. The complementary Stimulus Intensity Independent Pain Signature (SIIPS1) controls for stimulus intensity as well as the NPS and was applied to assess pain-related brain activations above and beyond nociceptive processing, for example, brain regions implicated in pain modulation [31]. Each signature produces a single score per individual, and group differences were analyzed with an independent *t*-test after having confirmed equality of variances using Levene’s test (NPS: *w* = 0.122, *p* = 0.727; SIIPS: *w* = 1.548, *p* = 0.217). The omnibus test of normality [32, 33] was implemented in SciPy [34]. Next, the correlation between the pain signatures was performed, using the robust skipped Spearman’s correlation [35, 36] with the Python package Pingouin [37] (version 0.3.8). Because of technical difficulties and problems with removal of metal objects (e.g., piercings) only 31 of 41 NSSI participants were scanned and included in the fMRI analysis.

## Results

### Participant characteristics

A total of 81 women, mean age 23.4 years (SD = 3.9), were included in the study (Table 1). For participants with NSSI, the age of self-injury onset was mean 13.2 years (SD = 3.1), and the average duration of self-harm was thus 10 years. The clinical characteristics displayed significant differences between NSSI participants and controls with NSSI participants reporting more borderline symptoms, self-critical cognitive style, and difficulties with emotion regulation.

**Table 1:** Demographic and clinical characteristics.

	Mean (SD)				
Characteristic	NSSI	Controls	Total	Statistic	*P* value
Number of participants	41	40	81		
Demographic characteristics					
Age, mean (SD), years	22.7 (3.6)	24.0 (4.1)	23.4 (3.9)	*t* = −1.54	0.128
Educational level, n (%)					
Elementary school	7 (17.1)	7 (17.5)	14 (17.3)	χ^2^ = 1.19	0.755
High school	27 (65.9)	24 (60.0)	51 (63.0)		
College degree	5 (12.2)	8 (20.0)	13 (16.1)		
Other	2 (4.9)	1 (2.5)	3 (3.7)		
Clinical characteristics					
BSL-23, mean (SD)	2.3 (0.9)	0.4 (0.3)	1.3 (1.2)	*t* = −13.0	<0.001
SRS, mean (SD)	33.7 (8.5)	16.2 (7.9)	25.0 (12.0)	*t* = −9.7	<0.001
DERS-16, mean (SD)	56.2 (13.3)	27.1 (9.9)	41.8 (18.8)	*t* = −11.2	<0.001

*SD* standard deviation, *NSSI* nonsuicidal self-injury, *BSL-23* Borderline Symptom List, *SRS* Self-Rating Scale (self-criticism), *DERS-16* 16 Item Version of the Difficulties in Emotion Regulation Scale.

Internal consistency for the questionnaires used in the NSSI group can be found in eTable 4 in Supplementary B.

Of the 41 participants with NSSI, 28 regularly used medication, compared with 4 of the controls. Most used medications among participants with NSSI was selective serotonin reuptake inhibitors (SSRIs), used by 19 of the 41 participants. Five participants with NSSI and two controls reported having taken as needed medications that could affect their pain regulation less than 24 h before the pain tests. All of them had taken the medication the day before the tests.

During the clinical interview 32 out of 39 replied that they had a psychiatric diagnosis. For two participants the response is missing. The most frequently reported diagnosis was depression (*n* = 21), followed by anxiety disorder (*n* = 12), and borderline personality disorder (*n* = 11).

Most common types of self-harm reported were hitting oneself on purpose (*n* = 32) and cutting or carving on the skin (*n* = 31). Mean number of methods used was 5.6 (SD = 2.2), range 2–12. The most reported reasons for self-harm were “to punish yourself” and “to stop bad feelings”.

### Pain tests

#### Heat pain thresholds and tolerance

**Fig. 2:**
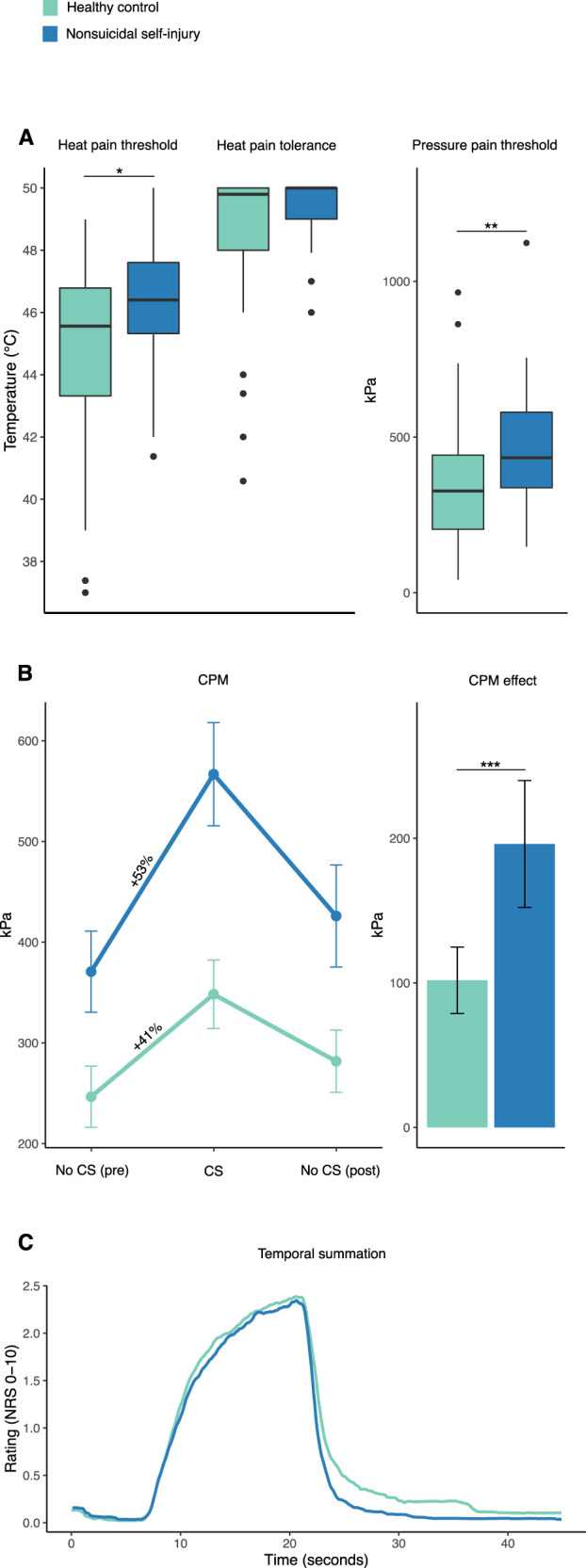
Results from pain testing. **A** Boxplots (including median, interquartile range, and upper and lower adjacent values) of heat pain threshold, heat pain tolerance, and pressure pain threshold. **B** Mean pressure pain threshold during CPM procedure and CPM effect (error bars represent 95% confidence interval). **C** Mean pain ratings during repeated stimulation in the temporal summation procedure. Abbreviations: CPM conditioned pain modulation, CS conditioning stimulus. *P < 0.05, **P < 0.01, ***P < 0.001.

Model estimated heat pain threshold was 46.3 °C (SD = 2.9) for the NSSI group and 44.8 °C (SD = 2.1) for the control group (Fig. 2A). There was a significant difference between the groups, *P* = 0.031. Model estimated heat pain tolerance was 49.3 °C (SD = 2.4) for the NSSI group and 48.5 °C (SD = 1.0) for the control group (Fig. 2A). There was no significant difference between the groups, *P* = 0.192.

#### Pressure pain

Mean pressure pain threshold was 463 kPa (SD = 189) for the NSSI group and 356 kPa (SD = 202) for the control group (Fig. 2B). There was a significant difference between the groups, *P* = 0.007.

#### Conditioned pain modulation

Model estimated conditioned pain modulation response was 196 kPa for the NSSI group and 102 kPa for the control group (Fig. 2C, D). There was a significant difference between the groups, *P* < 0.001.

#### Temporal summation

Model estimated facilitation of pain was NRS 1.9/10 for the NSSI group and NRS 1.8/10 for the control group (Fig. 2E). There was no significant difference between the groups, *P* = 0.723.

Correlational analyses between pain outcomes, self-report and clinical data

We found no correlations between the outcomes from pain testing, self-report data of NSSI behavior or clinical characteristics (Table 2).

**Table 2:** Correlations between pain measures and self-report data.

	Pressure threshold	Heat threshold	CPM effect	NSSI frequency	Severe NSSI methods	NSSI recency	NSSI duration	BSL	SRS	DERS
Pressure threshold										
Heat threshold	0.1									
CPM effect	0.13	0.22*								
NSSI frequency	−0.19	−0.23	−0.05							
Severe NSSI methods	−0.19	0.08	−0.15	0.13						
NSSI recency	0.07	0.09	−0.04	−0.21	−0.09					
NSSI duration	0.06	0.01	−0.04	−0.14	−0.16	−0.16				
BSL	0.03	−0.15	−0.08	0.15	0.09	−0.06	−0.01			
SRS	−0.06	−0.11	0	0.28*	0.09	−0.22*	−0.12	0.35**		
DERS-16	−0.02	−0.17	−0.06	0.28*	0.1	−0.06	−0.16	0.5***	0.38***	

*CPM* conditioned pain modulation, *NSSI* nonsuicidal self-injury, *BSL* Borderline Symptom List, *SRS* Self-Rating Scale (self-critical cognitive style), *DERS-16* 16 Item Version of the Difficulties in Emotion Regulation Scale.

*P < 0.05; **P < 0.01; ***P < 0.001.

#### fMRI

**Fig. 3:**
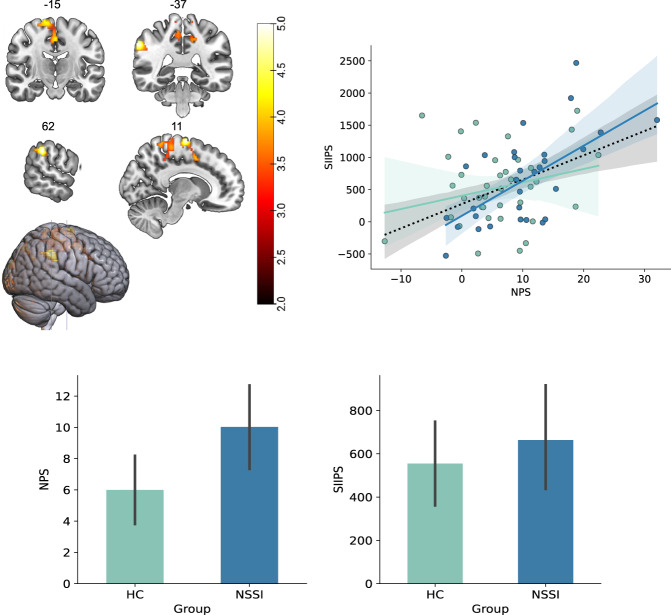
Brain responses during nociceptive processing. **A** Brain regions where NSSI had higher neural activations than controls during calibrated (moderate) heat pain. Significant activations are located in the leg area of the primary somatosensory cortex. A statistical threshold of *P* < 0.05 FWE-corrected for multiple comparisons across the entire brain was used, and the heat bar represents z-values. **B** The neurologic pain signature (NPS) responses during nociceptive processing were higher in NSSI individuals compared to controls. The stimulus-intensity independent pain signature (SIIPS1) responses, representing extranociceptive brain regions (eg, pain modulatory) were not different between groups. **C** Correlations between signatures. There was a significant correlation between nociceptive and extra-nociceptive brain responses to pain in NSSI individuals, but not in controls. Error bars represent 95% confidence interval.

In our analysis of brain activations during moderate thermal pain, NSSI participants had increased neural activations in the leg area of the primary somatosensory cortex (S1) (MNI coordinates: *x* = 11, *y* = −10, *z* = 72; z-score = 4.63, *P* = 0.022) and secondary somatosensory cortex (S2) (MNI coordinates: *x* = 62, *y* = −37, *z* = 37; z-score = 4.68; *P* = 0.013), contralateral to the stimulation site (Fig. 3A). There were no regions where controls had greater neural activations than NSSI participants. The individual calibration of heat pain was successful as the mean pain rating (NRS) during fMRI scanning did not differ between groups; 4.5/10 (SD = 1.8) for NSSI and 4.6/10 (SD = 2.0) for the control group.

Both the NPS and SIIPS brain signatures were used to assess differences in brain activations between NSSI and controls, as the two signatures are complimentary and based on different brain circuitry. A between-group comparison of NPS outcomes revealed augmented pain processing in nociceptive brain areas in NSSI individuals (Fig. 3B) (independent *t*-test, *T* = 2.238, *p* = 0.028). There was no difference between NSSI individuals and controls in extra-nociceptive pain processing areas, as determined by SIIPS (Fig. 3B) (*T* = 0.704, *p* = 0.483).

A correlation between NPS and SIIPS values showed a significant correlation across both groups (*r* = 0.316, CI 95% = [0.08, 0.52], *R*^*2*^ = 0.100, adjusted *R*^*2*^ = 0.072, *p* = 0.009). When unpacking the result, the correlation was only significant in the NSSI group (*r* = 0.489, CI 95% = [0.16, 0.72], *R*^*2*^ = 0.239, adjusted *R*^*2*^ = 0.183, *p* = 0.008, with two outliers). The corresponding result for controls was *r* = 0.115, CI 95% = [−0.21, 0.42], *R*^*2*^ = 0.013, adjusted *R*^*2*^ = −0.043, *p* = 0.491, with no outliers (Fig. 3C).

## Discussion

Despite a marked inability to regulate negative emotions, the present results provide evidence for enhanced pain regulation among individuals with NSSI, as indicated by conditioned pain modulation. The results indicate that NSSI is not associated with a general lack of inhibitory control, as might be inferred from the pronounced problems with emotion regulation. Instead, our data provide evidence for augmented inhibition of pain-specific signals, reflected in altered neural representations in nociceptive brain regions.

### Pain testing

In line with previous research [12, 13] we found higher heat- and pressure pain thresholds in NSSI, indicating more effective pain regulation. To reveal the underlying mechanisms, tests of pain inhibitory control and facilitation were performed. In line with our hypothesis, conditioned pain modulation was stronger in NSSI individuals. Contrary to our pre-specified hypotheses, temporal summation of pain, assessing facilitation of pain signals, was not different between groups. Taken together, we conclude that the differences in pain sensitivity between NSSI and controls is likely represented by inhibitory, rather than facilitatory, neural processes.

### Brain imaging results

Results from our brain imaging analyses revealed differences in nociceptive processing between NSSI individuals and controls. During the painful blocks of heat, participants with NSSI had increased activations in somatosensory brain regions compared to controls, including the leg area of S1 contralateral to the pain stimulation. One way to interpret this finding is that NSSI participants had a relatively greater involvement of somatosensory brain circuits during noxious input, as opposed to brain circuits related to salience and motivational value. As negative affect is likely to worsen pain, the increased somatosensory involvement may be significative of less fear-related amplification of pain, and potentially facilitate activation of pain inhibitory responses. Previous data among healthy volunteers show increased activity in S1 during increased attention to a painful stimulus, and attenuation of S1 during self-administered pain [38]. This could potentially reflect the differences between NSSI participants and controls in S1 seen here, as the pain was induced by the experimenter and thus represent a situation different from the habitual self-injury. As we used perception-matched heat stimuli, the temperatures given to the leg were slightly higher in the NSSI group, which may partly explain (but not fully) the increased S1 and S2 activations independent from the marked difference in pain perception (see Supplementary B). The NPS analysis corroborated our initial group comparison (univariate analysis) by indicating higher NPS scores in NSSI individuals compared to controls. Our second pain signature, SIIPS, includes brain regions involved in modulation of pain and emotional processing (eg, dlPFC, caudate, ventrolateral PFC (vlPFC), Nucleus Accumbens (NAc) and parahippocampal cortex), and did not differ between NSSI and controls. However, the correspondence between NPS and SIIPS was greater for NSSI, indicating that the integration between nociceptive and extranociceptive brain circuitry may be key for understanding the pain insensitivity seen in NSSI.

### Clinical outcomes

Both repeated exposure to pain and an innate anti-nociceptive profile have been proposed as explanations to the pain insensitivity in NSSI [39]. In a cross-sectional study comparing patients with borderline personality disorder with ongoing and previous NSSI, the group with ongoing NSSI experienced higher pain thresholds [40] and Magerl et al. [41] found a correlation between recency of self-injury behavior and pressure pain threshold. However, the only study that have assessed pain in patients with NSSI longitudinally by Koenig et al. [42] showed no change in pain sensitivity over one year’s time, despite a significant decline in NSSI. In the current study, we observed no correlations between the experimental pain outcomes and NSSI characteristics, i.e., NSSI frequency during the last year, recency (number of days since last NSSI behavior), duration (years since NSSI debut), or use of severe methods. Thus, our results are in line with those of Koenig et al. [42] and also of Glenn et al. [43] who found no correlations between NSSI duration, or frequency (last month/year/lifetime) and pain sensitivity, which adds to the evidence that the anti-nociceptive pain profile seen in individuals with NSSI may be innate.

It has been suggested that borderline symptomatology [12, 14] or a self-critical cognitive style [15, 44], links NSSI and pain insensitivity. In our data, we found no correlations between the pain assessments and the assessments of borderline symptoms or self-critical cognitive style. Neither was there any correlation between the pain assessment and the assessment of emotion regulation. Emotion regulation is a type of central regulation in which individuals with NSSI typically deviate from controls. The NSSI participants in our study displayed augmented pain inhibition, while their ability to regulate emotions was significantly impaired. This is in agreement with previous studies suggesting that pain and emotion regulation are partly distinct and uncorrelated systems [45].

### Clinical relevance

Knowledge of what aspect of the pain regulation is augmented in patients with NSSI can be used to inform medical and behavioral treatment targets [46]. According to the Benefits and barriers model by Hooley and Franklin [11], what differentiate self-injurers from non-self-injurers is not the benefits of NSSI, but rather the diminished barriers to NSSI, and that is why new treatments should focus on the barriers to NSSI. Pain is likely one of the most important barriers that stops people from engaging in NSSI. For individuals with NSSI, it may be possible to reinstate pain as a barrier with the help of behavioral or pharmacological interventions. For patients, knowledge of the pain’s role in NSSI may increase understanding and help motivate behavioral change. Also, pain profiling of psychiatric patients may help identify individuals at risk of developing NSSI and suicidal behaviors.

### Limitations

A limitation to our study is the case–control study design, which precludes any causal conclusions regarding NSSI exposure and pain characteristics. Another limitation is the lack of control for psychiatric comorbidity. However, it is not likely that the psychiatric burden accounted for the pain insensitivity in the participants with NSSI. In fact, a large study comparing the pain sensitivity of 735 patients with depressive disorder (the most common psychiatric comorbidity of the participants with NSSI in our study) with 456 healthy controls conclude that patients with depression were more sensitive to pain [47]. Another limitation is the ceiling effect for heat tolerance (Fig. 2A), impeding the comparison between the groups. A previous NSSI study by Leone et al found conflicting results, in which NSSI participants showed weaker conditioned pain modulation compared to controls. This may be explained by differences in the population being tested (adolescents) or the stimulus modality being used (heat). NSSI is highly prevalent in adolescents and this group is likely more heterogeneous than adults with NSSI. It is also possible that heat is not equally relevant to NSSI pain as pressure pain. The results of the present study are in line with those by Defrin et al in patients with borderline personality disorder [20]. However, differences in pain modulation between patients with and without NSSI could not be demonstrated in the Defrin study, possibly because of a small sample size. Strengths of the study include the careful application of well-established pain tests, the rigorous inclusion procedure, mechanistic approach by adding brain imaging, and its pre-registration.

## Conclusions

We found a more effective central down-regulation of pain in participants with NSSI compared with controls, while facilitation of pain signals did not differ between the groups. Also, the neural responses to painful stimulation revealed a stronger relation between nociceptive and pain modulatory brain regions in NSSI compared to controls. The augmented pain inhibition and higher involvement of pain modulatory brain networks in NSSI may represent a signature of greater risk for developing NSSI and suicidal behaviors.

## Supplementary information

Supplementary A: Methods

Supplementary B: Results
