# Supplementary material for: Augmented pain inhibition and higher integration of pain modulatory brain networks in women with self-injury behavior
Source: Mol Psychiatry. 2022 Jun 13;27(8):3452–9. doi: 10.1038/s41380-022-01639-y (PMC9708552; doi:10.1038/s41380-022-01639-y)
Supplement: Supplementary file 1 — Supplementary A: Methods [file 41380_2022_1639_MOESM1_ESM.docx]

## Supplementary A: Methods

**Pain calibration**

During pain calibration were exposed to between 13-24 heat stimulations (depending on the pain sensitivity of the participant). Each heat stimulation lasted 5 seconds, with an interstimulus interval of 35 seconds. Participants used a trackball to rate their pain intensity on a 0-10 NRS that was displayed on a screen after each stimulation. The heat pain calibration included four blocks of stimulations. In the first block, the first stimulation was 38°C. The temperature was then raised 1°C for each subsequent stimulation, until the participants rated their pain above NRS 1/10. In the second block, participants were given four stimulations starting from -1°C of the last temperature given in first block. For example, if the first block ended with 45°C, the participants were given a stimulation sequence of 44°C, 45°C, 46°C, 47°C, in a randomized order. The third and the fourth block, the stimulation sequence in block were once again given in a randomized order, but depending on the participants’ rating in the previous block the overall temperature was modified. If the maximum pain rating during the previous block did not exceed NRS 3/10 the overall temperature of the next stimulation sequence was raised 3°C: [44°C, 45°C, 46°C, 47°C] ⇒ [47°C, 48°C, 49°C, 50°C]. If the maximum pain rating during the previous block exceeded NRS 6/10 the overall temperature of the next stimulation sequence was lowered 1°C: [44°C, 45°C, 46°C, 47°C] ⇒ [43°C, 44°C, 45°C, 46°C]. If the max maximum pain rating during previous block was between NRS 3/10 - NRS 6/10, the temperature was raised 1°C: [44°C, 45°C, 46°C, 47°C] ⇒ [45°C, 46°C, 47°C, 48°C]. The temperature never exceeded 50°C, to avoid the risk of tissue damage. Heat pain threshold and heat pain tolerance were calculated by fitting a linear regression to each participant’s pain ratings during the last three blocks of the pain calibration (see Statistical analysis: Behavioral data in main article).

**Neuroimaging data preprocessing**

Data quality was reviewed using MRIQC (https://mriqc.readthedocs.io/en/stable/ ). Frame-wise displacement was used to determine if there was excessive head motion from one volume to the next. No participants had to be excluded from the analyses, as none displayed a frame-wise displacement of >0.5 in >15% of the images. The fmriprep pipeline for data preprocessing was used (<https://fmriprep.org/>), including realignment, and normalization to Montreal Neurological Institute (MNI) space. Images were then spatially smoothed using 8-mm full-width half-maximum Gaussian kernel. Data analyses were performed using Statistical Parametric Mapping 12 (SPM12), and Matlab2014 (The MathWorks, Inc, Natick, MA).

**Exploratory region of interest analysis**

To further explore relevant areas for top-down modulation of pain we performed a region of interest (ROI) analysis restricted to the bilateral insula, bilateral anterior cingulate (ACC) and bilateral amygdala from AAL (Automated Anatomical Labeling) [1]. In addition, we selected bilateral dorsolateral prefrontal cortex dlPFC (Brodmann area BA9 and BA46), and periaqueductal grey (PAG) thresholded at 25% [2]. ROIs were combined in marsbar [3]. We compared groups (NSSI > HC, HC > NSSI) correcting for family-wise error (FWE) as well as at an uncorrected p-value of 0.001.

**References**

1. Tzourio-Mazoyer N, Landeau B, Papathanassiou D, Crivello F, Etard O, Delcroix N, et al. Automated Anatomical Labeling of Activations in SPM Using a Macroscopic Anatomical Parcellation of the MNI MRI Single-Subject Brain. Neuroimage. 2002;15:273–289.

2. Ezra M, Faull OK, Jbabdi S, Pattinson KT. Connectivity‐based segmentation of the periaqueductal gray matter in human with brainstem optimized diffusion MRI. Hum Brain Mapp. 2015;36:3459–3471.

3. Brett M, Anton J-L, Valabregue R, Poline J-B. Region of interest analysis using an SPM toolbox. J Neurol Sci. 2017;381:14.
