# Supplementary material for: Augmented pain inhibition and higher integration of pain modulatory brain networks in women with self-injury behavior
Source: Mol Psychiatry. 2022 Jun 13;27(8):3452–9. doi: 10.1038/s41380-022-01639-y (PMC9708552; doi:10.1038/s41380-022-01639-y)
Supplement: Supplementary file 2 — Supplementary B: Results [file 41380_2022_1639_MOESM2_ESM.docx]

## Supplementary B: Results

**Conditioned pain modulation**

In accordance with the preregistered mixed effects model, order was specified as a fixed effect and by-subject random effect (model1 in eTable 1). Because of the high correlation between the effect of order and condition, the model did not converge. For that reason, we decided to drop order as a fixed and random effect. The results of the second model (model2 in eTable 2), without order as fixed and random effect, was reported in the main article. Visual inspection of diagnostic plots (eFigure 1) of the second model suggested that the assumption of homoscedacity and normality had been violated, possibly due to the large variance in the CPM effect in the NSSI group, that on average displayed higher CPM effect. Therefore, we also compared the mean CPM effect (difference between pain threshold, before and during conditioned stimulus) of the two groups, using non-parametric Wilocoxon rank sum test. We found a significant difference between the groups, *P* < .001. We also constructed a model without the participants who reported taking SSRI or SNRI medication, including 20 participants in the NSSI group. The model estimated conditioned pain modulation response was 162 kPa for the NSSI group and 102 kPa for the control group. There was a significant difference between the groups, *P* = .018. The code that accompanies these results can be found in ‘cpm_results.R’ in the R-script folder on https://osf.io/gujwt/. We adapted eTable 2 and 3 from from Appendix 5 in Meteyard & Davies [1].

eFigure 1. Conditioned pain modulation: diagnostic plots of mixed effects model (model2)
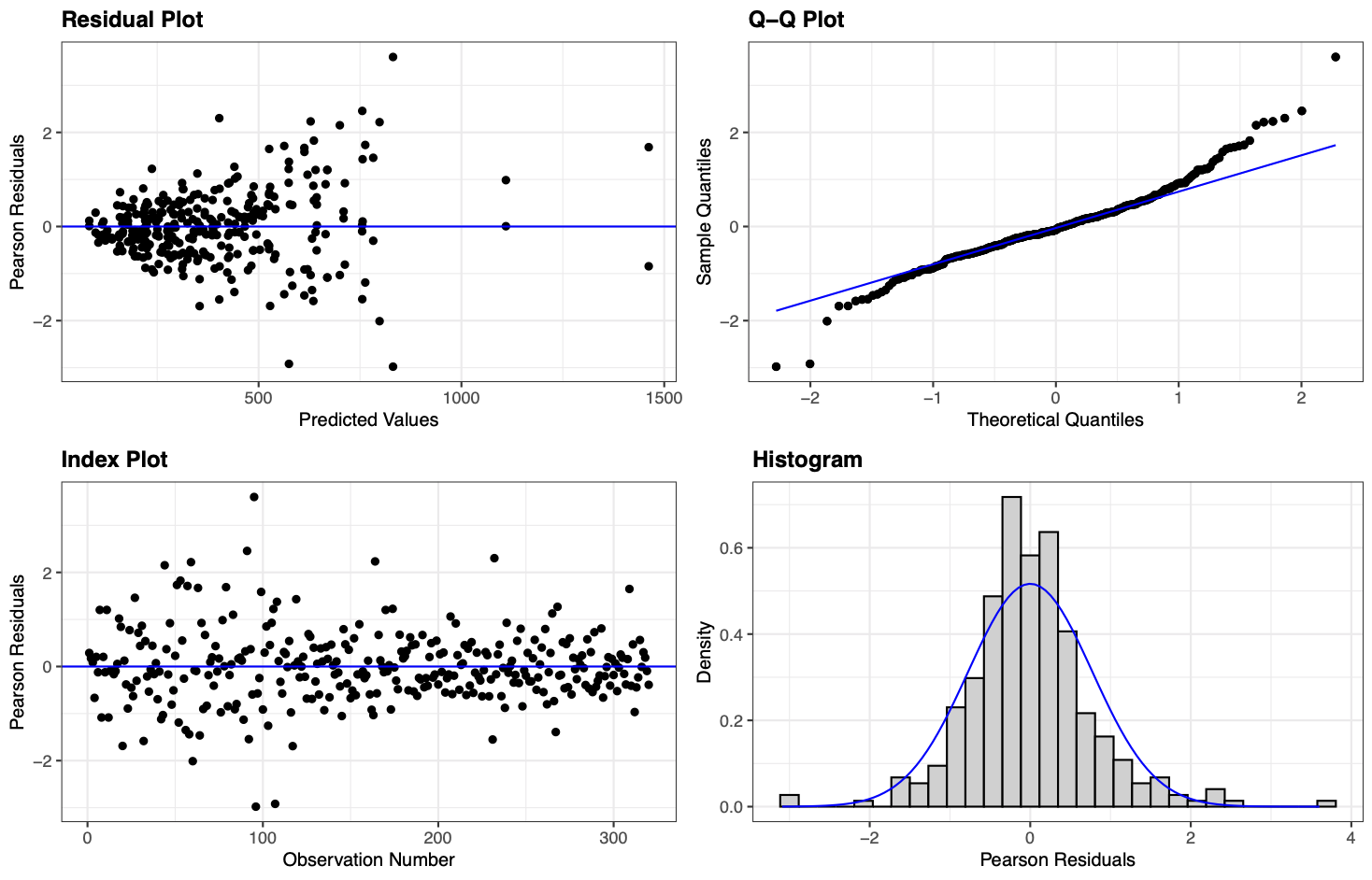


eTable 1. Conditioned pain modulation: model selection

| Model specification | Model name | Fixed Effects | Random Effects | Error/ Warning | Model fit | | |
| --- | --- | --- | --- | --- | --- | --- | --- |
|  |  |  |  |  | AIC/BIC | LL | df.resid |
| Linear model: pre-registered model | model1 | Condition*Group + Order | Condition + Order \| Subject | Model convergence problem; singular convergence | 3910.1/3955.3 | -1943.1 | 308 |
| Linear model: exclude order | model2 | Condition*Group | Condition \| Subject |  | 3916.6/3946.7 | -1950.3 | 312 |
| Linear model: exclude SSRI/SNRI | model3 | Condition*Group | Condition \| Subject |  | 2821.9/2849.7 | -1403.0 | 228 |

eTable 2. Condition pain modulation: model estimates for the mixed effects model (model2)

| Fixed effects | | | | | |
| --- | --- | --- | --- | --- | --- |
|  | Est/Beta | SE | 95% CI | df, *t* | *P* |
| Intercept | 246.42 | 25.78 | 195.28 - 297.60 | 80, 9.56 | <0.001 |
| Condition_CS_ | 101.78 | 17.58 | 66.90 - 136.67 | 80, 5.79 | <0.001 |
| Group_NSSI_ | 124.21 | 36.01 | 55.77 - 195.65 | 80, 3.45 | <0.001 |
| Condition_CS_ × Group_NSSI_ | -94.32 | 24.56 | 45.59 - 143.04 | 80, 3.84 | <0.001 |
|  | | | | | |
| Random effects | | | | | |
|  | Variance | SD | Correlation | | |
| Subject (intercept) | 24050 | 155.08 |  | | |
| Subject × Condition_CS_ (Slope) | 8309 | 91.15 | 0.10 | | |
| Residual | 3751 | 61.24 |  | | |
| *Note*. P-values for fixed effects were calculated using Satterthwaites approximations. Model formula: kPa ~ Condition * Group + (Condition \| Participant). N = 80, total observations = 320 | | | | | |

**Temporal summation**

The code that accompanies the results presented in eTable 3 can be found in ‘tempsum_results.R’ in the R-script folder on https://osf.io/gujwt/.

eTable 3. Temporal summation: model estimates for the mixed effects model

| Fixed effects | | | | | |
| --- | --- | --- | --- | --- | --- |
|  | Est/Beta | SE | 95% CI | df, *t* | *P* |
| Intercept | 0.87 | 0.09 | 0.69 - 1.05 | 76.61, 9.51 | < 0.001 |
| Stimulus_max_ | 1.77 | 0.19 | 1.40 - 2.15 | 76.75, 9.34 | < 0.001 |
| Group_NSSI_ | -0.05 | 0.13 | -0.31 - 0.22 | 76.19, -0.34 | 0.734 |
| Stimulus_max_ × Group_NSSI_ | 0.10 | 0.28 | -0.45 - 0.65 | 76.51, 0.36 | 0.723 |
|  | | | | | |
| Random effects | | | | | |
|  | Variance | SD | Correlation | | |
| Subject (intercept) | 0.25 | 0.50 |  | | |
| Subject × Stimulus_max_(slope) | 1.28 | 1.13 | 0.38 | | |
| Residual | 0.30 | 0.55 |  | | |
| *Note*. P-values for fixed effects were calculated using Satterthwaites approximations.  Model formula: Rating ~ Stimulus * Group + (Stimulus \| Participant). | | | | | |

**fMRI**

Control for stimulus intensity (temperature)

To estimate the contribution of differences in stimulus intensity on pain-related brain activations an exploratory t-test was performed, using each individual’s calibrated temperature as covariate in the analysis. The result yielded activations similar to the analysis performed without the covariate, with slightly reduced z-scores in the peak voxels of the two significant clusters, i.e. S1 (z-score= 4.04) and S2 (z-score=4.02), respectively. The clusters did not survive FWE-correction for multiple comparisons.

Neurological Pain Signature

When restricting the analysis to a priori defined pain regions, using the Neurologic Pain Signature (NPS) template, NSSI had higher activations in three clusters compared to controls: mid cingulum (x = 2, y = 6, z = 44, t = 4.02, *P* = .014), anterior cingulum (x=-5, y=21, z=30, t=3.58, p=.037), and the anterior insula (x = 40, y = 11, z = 2, t = 3.55, *P* =.037). There were no regions where HC had greater neural activations within the NPS than NSSI.

Exploratory region of interest analysis

An exploratory region-of-interest (ROI) analysis of selected brain regions implicated in top-down modulation of pain showed higher involvement of the dorsomedial prefrontal cortex (dlPFC), Anterior Cingulate Cortex, and insula in NSSI individuals compared to controls (*P* = .001) in line with our hypothesis and behavioral results. There were no statistically significant results in the amygdala and PAG. However, the ROI analysis was not defined *a priori* and did not survive correction for multiple comparisons and should therefore be interpreted with caution.

eTable 4. Two-sided paired t-test comparing NSSI and HC for pain versus no pain. Results are uncorrected at p<0.001.

| ROI | *T* | MNI (X, Y, Z) |
| --- | --- | --- |
| L dlPFC | 3.91 | -37, 52, 23 |
| L ACC | 3.80 | -8, 21, 30 |
| L Insula | 3.58 | -34, 9, 12 |
| R Insula | 3.55 | 40, 11, 2 |
| R dlPFC | 3.46 | 28, 47, 20 |
| R Insula | 3.46 | 35, -13, 6 |
| R Insula | 3.35 | 50, 11, -8 |
| R Insula | 3.32 | 38, 9, -12 |
| R Insula | 3.22 | 38, -6, 6 |
|  |  |  |

eFigure 2. Two-sided paired t-test comparing NSSI and HC for pain versus no pain. Results are uncorrected at p<0.001.


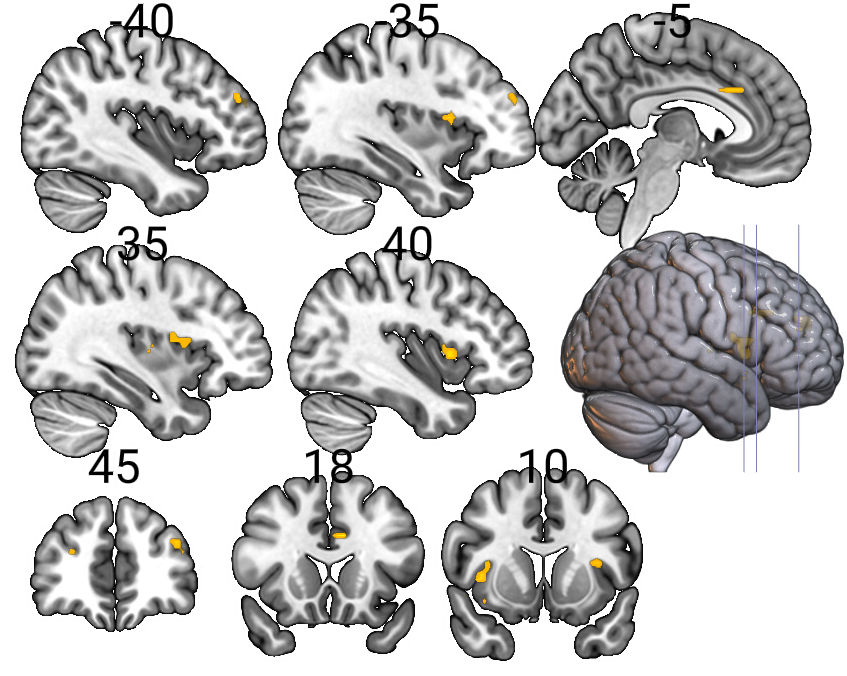


**Questionnaires**

Internal consistency for the questionnaires assessed by NSSI participants are presented in eTable 4.

eTable 4. Internal consistency for the questionnaires used in the NSSI group (n=41)

|  | Number of items | Cronbach’s α |
| --- | --- | --- |
| Borderline Symptom List | 23 | .95 |
| Self-Rating Scale | 8 | .82 |
| Difficulties with Emotion Regulation Scale | 16 | .93 |

**References**

1. Meteyard L, Davies RA. Best practice guidance for linear mixed-effects models in psychological science. J Mem Lang. 2020;112:104092.
